# A Pocketful of Reminiscences

**Published:** 1914-01-31

**Authors:** 


### HOSPITALS FROM THE PATIENTS' POINT OF VIEW.

(Contributions to this Section are Invited.)

### A Pocketful of Reminiscences.

By "AITCHELL."

My first experience of the hospital was gained whilst a patient in a well-known London ophthalmic institution. After ten weeks of suffering with iritis following conjunctivitis I was advised, because the trouble could not be remedied by home treatment, to go into hospital. There I found that the most obstinate pupil will dilate with careful attention and the assistance of atropin, hot-boxes, leeches, blisters, and the like. My first night in hospital was sleepless for two reasons. First, I was not used to the noise caused by the heavy traffic constantly passing in the street below; and, secondly, I had grave fears that some description of operation was necessary. Being untutored I noticed with no small amount of apprehension that my temperature was one point above the normal on the following morning; but upon carefully questioning other patients I learned that "another bloke wot 'ad 'irittis' never 'ad no operation." After this information I slept the sleep of the satisfied; my temperature chart being, from the nurse's point of view, very uninteresting. Bohemian by nature I found the hospital day—from 5.30 a.m. until 8 p.m.—a long one; and had it not been for the kindness of the nurses I should have perished from another disease—*ennui.* I believe that the majority of women who devote themselves to hospital nursing are the highest principled of the sex. In this particular institution, carefulness, sound common sense, and cleanliness prevailed. My opposite bedmate (designated "the Count") had travelled in most countries, was sixty-four years of age, and a bachelor. Although in these advancing years and suffering from senile cataract I found him good company; his various intellectual discourses on subjects so far apart as Bachelor's Buttons and Woman's Suffrage being a treat to listen to. As an instance of conversational variety let me mention a question which was wrestled with for days: "Why do Peers Marry Actresses in Preference to Hospital Nurses?" and the heated discussion, in which the nurses joined, upon the writer opining that whether a woman was an actress or a nurse was due to accident of birth.

The advent of a red-haired youngster in the ward was the signal for much weeping and gnashing of teeth. A little Bedouin—the nurse brought him in fast asleep, and upon awaking he had necessarily to be washed. The row that followed baffles description; screamingly urgent calls for his "Muvver" coming close upon each other. After a conciliatory discussion with the Sister, he consented to quieten down; and a few minutes later his board was placed at the head of his bed, upon which it was shown that—"Rufus Absolom, aged 9 years, whilst in the school playground, was hit by a stone, thrown at, and intended for, a boy other than Rufus." (Here followed diagrams showing position of wound and stating that anterior chamber was full of blood.)

Later on, Rufus obliged us with a rather different chorus—Ragtime, *in extenso*; and had he not been born such a scamp I feel certain his eye would have been saved.

Another interesting patient was an old pensioner, who constantly alleged that "there was no sugar in his tea." It is on record that the old gentleman once plaintively voiced the same remark upon tasting his broth at supper-time.

Whilst in hospital I became notorious because of my siffluistic ability. It appears that the House Surgeon had a failing in the same direction—"Hitchy-Koo" being his *forte* (as well as mine). For this reason the Sister, imagining the House Surgeon in the vicinity when elsewhere, was more frequently perturbed than she need have been, and I had it suggested that I should "koo" something different from "Hitchy." However, I understand that in another ward general regret was expressed when it was rumoured: "That feller wot whistles is going 'ome to-day."

